# Complete Genome Sequence of *Aggregatibacter actinomycetemcomitans* Strain IDH781

**DOI:** 10.1128/genomeA.01285-16

**Published:** 2016-11-10

**Authors:** Anthony C. May, Rachel L. Ehrlich, Sergey Balashov, Garth D. Ehrlich, Mayilvahanan Shanmugam, Daniel H. Fine, Narayanan Ramasubbu, Joshua Chang Mell, Carla Cugini

**Affiliations:** a Department of Oral Biology, Rutgers School of Dental Medicine, Newark, New Jersey, USA; b Department of Microbiology and Immunology, Center for Genomic Sciences (CGS), Institute for Molecular Medicine & Infectious Disease, Drexel University College of Medicine, Philadelphia, Pennsylvania, USA; c Genomics Core Facility, Clinical and Translational Research Institute (CTRI), Drexel University College of Medicine, Philadelphia, Pennsylvania, USA

## Abstract

We report here the complete genomic sequence and methylome of *Aggregatibacter actinomycetemcomitans* strain IDH781. This rough strain is used extensively as a model organism to characterize localized aggressive periodontitis pathogenesis, the basic biology and oral cavity colonization of *A. actinomycetemcomitans*, and its interactions with other members of the oral microbiome.

## GENOME ANNOUNCEMENT

*Aggregatibacter actinomycetemcomitans* is a Gram-negative nonmotile facultative anaerobe of the oral microbiota implicated in the development of localized aggressive periodontitis (LAP) (1, 2). Despite the lack of a complete genomic sequence, IDH781, a rough strain, is commonly used as a model for pathogenesis and basic bacteriology studies (3–11). Rough strains of *A. actinomycetemcomitans* display the classic star-shaped colony morphology observed in clinical isolates from LAP patients, so they are more appropriate for the study of *A. actinomycetemcomitans* biology than those displaying a smooth colony phenotype (12, 13).

IDH781 was grown under anaerobic conditions (10% hydrogen, 10% carbon dioxide, and 80% nitrogen) on brain heart infusion agar supplemented with glucose, sodium bicarbonate, and yeast extract. The rough phenotype was confirmed microscopically. Cells were scraped from agar plates and lysed in 625 µg/ml proteinase K, 1.25 mg/ml lysozyme, and 2% sodium dodecyl sulfate. Genomic DNA was purified by phenol-chloroform/isoamyl extraction (14). DNA integrity was verified by agarose gel electrophoresis, purity was evaluated spectrophotometrically, and concentration was determined fluorometrically.

Sequencing libraries were constructed using the Pacific Biosciences 20-kb template preparation protocol and Sage Science’s BluePippin size-selection system with a 5-kb fragment size cutoff. Pacific Biosciences single-molecule real-time (SMRT) sequencing was performed on an RSII instrument using a single SMRT cell with P6-C4 chemistry and a 3-h movie, producing 7,601 polymerase reads (*N*_50_, 11,624 nucleotides [nt]) and 153,117 postfiltered subreads (*N*_50_, 6,741 nt). *De novo* assembly with the HGAP assembler (version 2.3) yielded a single contig supported by a mean coverage of 283-fold (15).

The genome was circularized by permutation to start at the *dnaA* gene and remove terminal duplications using Circlator (version 1.0.2), followed by resequencing using RS_Modification_and_Motif_Analysis (version 2.3) to correct errors at the original contig break and to detect DNA methylation motifs based on significant interpulse duration signals (15, 16). Two well-supported m6A motifs were detected, G**A**TC and AGG**A**G (bold indicates the methylated residue), with >98% of motifs in the genome detected. Seven other unique nonpalindromic modifications were detected at low frequency; these consisted of 2 putative m6A motifs (34% and 30%), 1 putative m4C motif (11%), and 4 unknown base modifications (20%, 15%, 8%, and 4%). The significance and importance of these low-frequency interpulse duration signals remain unknown.

The final assembly was 2,291,252 bp, with a G+C content of 44.3%, consistent with other completed *A. actinomycetemcomitans* genomes. To verify strain identity and detect possible horizontal gene transfer events, genomic intervals were taxonomically assigned using Taxator-tk (version 1.3.1e) with the nonredundant-microbial_20140513 database (refpack from http://research.bifo.helmholtz-hzi.de/software) (17). All classified regions (39.8% of the genome) were assigned to the species *A. actinomycetemcomitans*.

Annotation was performed by NCBI using the Prokaryotic Genome Automated Annotation Pipeline (PGAAP, best-placed reference protein; GeneMarkS+; version 3.3) (18). The chromosome contains 2,206 genes, with 2,129 coding sequences, 19 rRNAs, and 54 tRNAs for all 20 amino acids plus selenocysteine, 4 noncoding RNAs (ncRNAs), and 3 predicted clustered regularly interspaced short palindromic repeats (CRISPRs).

### Accession number(s).

The complete genome sequence of *A. actinomycetemcomitans* strain IDH781 has been deposited in GenBank under the accession number CP016553. The version described here is the first version.

## References

[1] Slots J, Ting M 1999 *Actinobacillus actinomycetemcomitans* and *Porphyromonas gingivalis* in human periodontal disease: occurrence and treatment. Periodontol 2000 20:82–121. doi:10.1111/j.1600-0757.1999.tb00159.x. 10522224

[2] Slots J, Reynolds HS, Genco RJ 1980 *Actinobacillus actinomycetemcomitans* in human periodontal disease: a cross-sectional microbiological investigation. Infect Immun 29:1013–1020. 6968718 10.1128/iai.29.3.1013-1020.1980 PMC551232

[3] Shanmugam M, Gopal P, El Abbar F, Schreiner HC, Kaplan JB, Fine DH, Ramasubbu N 2015 Role of exopolysaccharide in *Aggregatibacter actinomycetemcomitans*-induced Bone resorption in a Rat model for periodontal disease. PLoS One 10:e0117487. doi:10.1371/journal.pone.0117487. 25706999 PMC4338281

[4] Haubek D, Poulsen K, Asikainen S, Kilian M 1995 Evidence for absence in northern Europe of especially virulent clonal types of *Actinobacillus actinomycetemcomitans*. J Clin Microbiol 33:395–401. 7714199 10.1128/jcm.33.2.395-401.1995 PMC227955

[5] Fine DH, Furgang D, Schreiner HC, Goncharoff P, Charlesworth J, Ghazwan G, Fitzgerald-Bocarsly P, Figurski DH 1999 Phenotypic variation in *Actinobacillus actinomycetemcomitans* during laboratory growth: implications for virulence. Microbiology 145:1335–1347. doi:10.1099/13500872-145-6-1335. 10411260

[6] Fine DH, Karched M, Furgang D, Sampathkumar V, Velusamy S, Godboley D 2015 Colonization and persistence of labeled and “foreign” strains of *Aggregatibacter actinomycetemcomitans* inoculated into the mouths of rhesus monkeys. J Oral Biol (Northborough) 2:10. doi:10.13188/2377-987X.1000005. PMC4511163 26213715

[7] Takashima E, Konishi K 2008 Characterization of a quinol peroxidase mutant in *Aggregatibacter actinomycetemcomitans*. FEMS Microbiol Lett 286:66–70. doi:10.1111/j.1574-6968.2008.01253.x. 18616592

[8] Yue G, Kaplan JB, Furgang D, Mansfield KG, Fine DH 2007 A second *Aggregatibacter actinomycetemcomitans* autotransporter adhesin exhibits specificity for buccal epithelial cells in humans and Old World primates. Infect Immun 75:4440–4448. doi:10.1128/IAI.02020-06. 17620359 PMC1951147

[9] Fine DH, Goncharoff P, Schreiner H, Chang KM, Furgang D, Figurski D 2001 Colonization and persistence of rough and smooth colony variants of *Actinobacillus actinomycetemcomitans* in the mouths of rats. Arch Oral Biol 46:1065–1078. doi:10.1016/S0003-9969(01)00067-X. 11543714

[10] Fine DH, Kaplan JB, Furgang D, Karched M, Velliyagounder K, Yue G 2010 Mapping the epithelial-cell-binding domain of the *Aggregatibacter actinomycetemcomitans* autotransporter adhesin Aae. Microbiology 156:3412–3420. doi:10.1099/mic.0.037606-0. 20688817 PMC3090143

[11] Kaplan JB, Velliyagounder K, Ragunath C, Rohde H, Mack D, Knobloch JK, Ramasubbu N 2004 Genes involved in the synthesis and degradation of matrix polysaccharide in *Actinobacillus actinomycetemcomitans* and *Actinobacillus pleuropneumoniae* biofilms. J Bacteriol 186:8213–8220. doi:10.1128/JB.186.24.8213-8220.2004. 15576769 PMC532409

[12] Preus HR, Sunday GJ, Haraszthy VI, Zambon JJ 1992 Rapid identification of *Actinobacillus actinomycetemcomitans* based on analysis of 23S ribosomal RNA. Oral Microbiol Immunol 7:372–375. doi:10.1111/j.1399-302X.1992.tb00639.x. 1284398

[13] Slots J 1982 Selective medium for isolation of *Actinobacillus actinomycetemcomitans*. J Clin Microbiol 15:606–609. 7068837 10.1128/jcm.15.4.606-609.1982 PMC272154

[14] Sambrook J, Russell DW 2006 Purification of nucleic acids by extraction with phenol:chloroform. Cold Spring Harb Protoc 2006:pii=pdb.prot4455. doi:10.1101/pdb.prot4455. 22485786

[15] Chin CS, Alexander DH, Marks P, Klammer AA, Drake J, Heiner C, Clum A, Copeland A, Huddleston J, Eichler EE, Turner SW, Korlach J 2013 Nonhybrid, finished microbial genome assemblies from long-read SMRT sequencing data. Nat Methods 10:563–569. doi:10.1038/nmeth.2474. 23644548

[16] Hunt M, Silva ND, Otto TD, Parkhill J, Keane JA, Harris SR 2015 Circlator: automated circularization of genome assemblies using long sequencing reads. Genome Biol 16:294. doi:10.1186/s13059-015-0849-0. 26714481 PMC4699355

[17] Dröge J, Gregor I, McHardy AC 2015 Taxator-tk: precise taxonomic assignment of metagenomes by fast approximation of evolutionary neighborhoods. Bioinformatics 31:817–824. doi:10.1093/bioinformatics/btu745. 25388150 PMC4380030

[18] Tatusova T, DiCuccio M, Badretdin A, Chetvernin V, Nawrocki EP, Zaslavsky L, Lomsadze A, Pruitt KD, Borodovsky M, Ostell J 2016 NCBI prokaryotic genome annotation pipeline. Nucleic Acids Res 44:6614–6624. doi:10.1093/nar/gkw569. 27342282 PMC5001611

